# Molecular Detection, Characterization, Antimicrobial Resistance and Genomic Epidemiology of Pathogenic Bacteria

**DOI:** 10.3390/antibiotics13060494

**Published:** 2024-05-27

**Authors:** Andrey Shelenkov

**Affiliations:** Central Research Institute of Epidemiology, Novogireevskaya Str., 3a, 111123 Moscow, Russia; fallandar@gmail.com

In recent decades, growing attention has been directed worldwide toward antimicrobial-resistant (AMR) bacterial pathogens causing infections in clinical, environmental, and food chain production settings. The healthcare-associated infections (HAIs) caused by multidrug (MDR)- or pandrug-resistant bacteria have already been declared a ‘silent pandemic’ [1] and have raised a global concern due to increased patient morbidity and mortality associated with such agents [2]. In order to develop advanced prevention measures and treatment plans to combat such infections, specialists in various fields, including microbiology, medicine, molecular biology, and bioinformatics, need to work together. This will ensure a better understanding of the mechanisms driving resistance development and dissemination within bacterial populations.

It is well known that resistance spreading in clinically relevant bacterial species like 

*Klebsiella pneumoniae* or *Acinetobacter baumannii* is usually driven by several lineages called ‘global clones’ or ‘international clones of high risk’, and thus the detection and surveillance of the isolates belonging to such clones represents an important epidemiological task [3,4]. In addition, the continuous monitoring of AMR gene presence within clinical, environmental, veterinary, and food samples is essential for tracking the underlying mechanisms of resistance acquisition and developing proper control measures, especially considering the One Health paradigm [5,6]. Currently, molecular and whole-genome sequencing (WGS)-based methods are the gold standard for global clone detection or investigations of the AMR genes, virulence factors, and plasmids for bacterial isolates, even in time-critical situations [7,8].

Given the above findings, this Special Issue was contemplated as a collection of high-quality papers describing various applications of molecular biology, WGS, and genomic epidemiology methods for investigations of bacterial pathogens, with additional focus on population structure and AMR gene detection.

The papers published in this Special Issue describe various aspects of antimicrobial resistance and whole-genome analysis of pathogenic bacteria. The first aspect includes the genomic analysis of particular *Escherichia coli* and *Klebsiella pneumoniae* strains, providing valuable insights into mechanisms of AMR acquisition and dissemination of the studied isolates (Contributions 2, 3, 4, and 10). The second aspect refers to more general molecular and genomic epidemiology studies elucidating the structure of bacterial populations of *K. pneumoniae*, *Acinetobacter*, non-*baumannii*, and other species in clinical and agricultural environments (Contributions 6, 7, 8, and 9). The third aspect pertains to the caveats within molecular diagnostics and infection prevention, namely, the detection of viable but non-culturable bacteria (contribution 5) and the revelation of potential probiotic agents against *Salmonella* infections (contribution 1). A brief description of all these reports is provided below.

Contribution 1 is dedicated to the detection of *Ligilactobacillus salivarius* bacterial strains, which can serve as a potential preventive probiotic against *Salmonella* infections in poultry, in particular, for the ones caused by *S*. Enteritidis, *S*. Infantis, and *S*. Kentucky. Since *Salmonella* is the leading cause of foodborne disease globally [9], the development of suitable prevention measures against its spread among poultry and other food-producing animals becomes crucial.

Contribution 2 reveals the possible mechanism of amikacin resistance in clinical isolates of *K. pneumoniae*. Hybrid long- and short-read WGS revealed the precise sequence of a small plasmid carrying an amikacin resistance gene in only one member of a group of isolates with identical cgMLST profiles and thus possessing very similar genome structure. This small plasmid made a difference in the AMR of these isolates, which was confirmed by susceptibility testing.

Contributions 3 and 4 involve the genomic analysis of *E. coli*. The former describes the diarrheagenic hybrid strain and includes a comparative analysis of its genome and plasmids with previous outbreak isolates. The latter reveals the possible mechanism of quinolone resistance in an MDR isolate. Although *E. coli* is usually considered to be less dangerous in a hospital than, for example, *K. pneumoniae*, it can also cause serious outbreaks [10] and thus requires special attention.

Contribution 5 proposes a method for the detection of nonculturable *Staphylococci* using bacteriophages. This approach facilitates effective distinction between viable and dead bacteria in a sample, which can be very beneficial since most bacterial analysis approaches rely on culturing methods and fail to produce accurate results for nonculturable strains [11].

Contribution 6 provides epidemiological data regarding the diversity and resistance gene presence for *Klebsiella spp*. samples isolated from dairy farms in Pakistan. The presence of multiple resistance genes, especially the ones encoding beta-lactamases CTX-M-15 and CTX-M-55, highlights the need for continued surveillance.

Contribution 7 involves a molecular analysis of beta-lactamase-producing *E. coli* and *Pseudomonas aeruginosa* in Pakistan tertiary care hospitals. The authors noted a general increase in AMR. Contribution 8 presents the in silico genomic epidemiology analysis of carbapenem-resistant *K. pneumoniae* (CRKP) in countries belonging to the Gulf Cooperation Council. The study revealed a wide distribution of high-risk clones and other epidemiologically valuable data.

Contribution 9 describes the wide-scale analysis of *Acinetobacter* non-*baumannii* species in hospitalized patients. Although most *Acinetobacter* studies are usually focused on *A. baumannii*, other members of this genus are becoming increasingly more important as opportunistic pathogens in clinical settings. The authors discuss the infections caused by these pathogens and the mechanisms of AMR, especially to carbapenem antibiotics.

Contribution 10 presents a genomic analysis of colistin-resistant *E. coli* isolates from camels and provides valuable insights into plasmid-mediated mechanisms of AMR transfer.

Taken together, the papers presented in this Special Issue highlight the importance of molecular, WGS, and genomic epidemiology methods for studying the clonal structure and AMR gene content within bacterial populations in clinical, environmental, and food production settings. In particular, WGS-based studies allowed researchers to capture the diversity of particular species and reveal possible AMR and virulence acquisition mechanisms in the studied isolates. Additionally, novel methods for *Staphylococci* detection and *Salmonella* spread prevention are presented.

The data provided in this Special Issue will be useful for researchers in various fields involving bacterial pathogen surveillance, detection, and treatment, especially in the emerging field of genomic epidemiology.
